# Time Trends in Cardiovascular Event Incidence in New‐Onset Type 2 Diabetes: A Population‐Based Cohort Study From Germany

**DOI:** 10.1111/1753-0407.70099

**Published:** 2025-06-02

**Authors:** Theresia Sarabhai, Karel Kostev

**Affiliations:** ^1^ Department of Endocrinology and Diabetology Medical Faculty and University Hospital Düsseldorf, Heinrich‐Heine‐University Düsseldorf Germany; ^2^ Epidemiology IQVIA Frankfurt am Main Germany; ^3^ University Clinic, Philipps‐University Marburg Germany

**Keywords:** cardiovascular disease, cerebrovascular events, coronary hearth disease, ischemic stroke, myocardial infarction, primary care, retrospective cohort study, temporal changes, transient ischemic attack, type 2 diabetes mellitus

## Abstract

**Background:**

Events of cardiovascular disease (CVD) remain a critical concern in patients with Type 2 diabetes mellitus (T2D). Over 17 years, this study analyzed time changes in the 5‐year incidence of myocardial infarction (MI), chronic coronary heart disease (CHD), transient ischemic attack (TIA), and ischemic stroke (IS).

**Methods:**

This retrospective cohort study was conducted using the Disease Analyzer database, including patients aged ≥ 18 years with at least 12 months of no prior CVD with new‐onset T2D in 2001–2006 (*n* = 10 162) and in 2013–2018 (*n* = 30 486), matched 1:3 by age and sex. Kaplan–Meier survival analysis estimated the 5‐year cumulative incidence of the outcomes. Multivariable Cox regression models assessed temporal changes, adjusted for comorbidities.

**Results:**

The incidence of CHD and TIA significantly declined in 2013–2018 compared to 2001–2006, with HRs of 0.68 (95% CI: 0.63–0.73; *p* < 0.001) and 0.63 (95% CI: 0.52–0.76; *p* < 0.001), respectively. Reductions were more pronounced in women and older patients. Surprisingly, MI incidence showed only a trend of reduction (HR: 0.82; 95% CI: 0.68–0.99; *p* = 0.045) and IS incidence was not different (HR: 0.97; 95% CI: 0.85–1.12; *p* = 0.722) between time periods.

**Conclusions:**

This study is the first to report time trends in CVD incidence in new‐onset T2D in Germany. From 2001 to 2018, the 5‐year incidence of CHD and TIA decreased in new‐onset T2D, reflecting demographic‐specific advancements in diabetes and cardiovascular care. However, the stable incidence of IS and MI underscores a persistent challenge in prevention strategies in patients with prediabetes and T2D.

## Graphical abstract

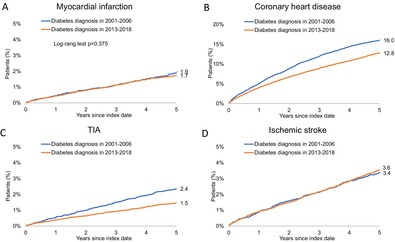

**Summary**

- This large retrospective cohort study from German primary care demonstrates that among patients with new diagnosed Type 2 diabetes and no prior cardiovascular disease, the 5‐year cumulative incidence of coronary hearth disease and transient ischemic attack declined from 2001‐2006 to 2013‐2018, especially in women and older adults.
- The effect was most pronounced in women and patients aged over 60 years.
- In contract, the incidence in myocardial infarction and ischemic stroke remained unchanged, possibly due to vascular damage during the pre‐diabetic phase, highlighting the need for earlier intervention.

## 1 Introduction

The importance of Type 2 diabetes (T2D) mellitus as a major cardiovascular and cerebrovascular risk factor and resulting diabetes‐related complications has received considerable attention in the last decades [1]. In Germany, the prevalence of T2D among individuals aged 18–79 years is 9.9%, with projections indicating a potential increase of up to 77% by 2040 [2, 3, 4]. Notably, T2D alone accounts for 16% of all deaths in Germany, predominantly due to cardiovascular causes, with the highest excess mortality observed among individuals aged 70–89 years [5]. The increase in the number of people with T2D or with a longer duration of T2D is likely to alter the disease profile and health‐care costs, particularly, due to a higher incidence of diabetes‐specific complications [6]. Newly diagnosed T2D amplifies the risk of cardiovascular disease (CVD) complications, including myocardial infarction (MI), chronic coronary heart disease (CHD), ischemic stroke (IS), and transient ischemic attack (TIA), by two‐ to fourfold, compared to the general population [7, 8]. The underlying mechanisms include chronic hyperglycemia, insulin resistance, systemic inflammation, and dyslipidemia, which collectively exacerbate vascular dysfunction and synergistically accelerate the progression of atherosclerosis [9].

In the preceding two decades, progress in the management of T2D has been marked by significant medical advancements, including enhanced glycemic control, earlier diagnoses, and the introduction of cardioprotective medications such as sodium‐glucose cotransporter‐2 inhibitors (SGLT2i) and glucagon‐like peptide‐1 receptor agonists (GLP‐1‐RA) [10, 11]. These advancements have led to a significant reduction in the incidence and mortality of CVD in both the general population and individuals with T2D [12, 13, 14, 15]. For instance, between 1988–1994 and 2010–2015, all‐cause mortality rates among US adults with diabetes fell by 20% per decade, and vascular‐related mortality dropped by 32% per decade [16]. Recent European guidelines even propose that individuals with short‐standing T2D (< 10 years) may not be at high cardiovascular risk [17]. The majority of preceding population‐based cohort studies have documented declines in cardiovascular events among individuals with T2D with long‐standing diabetes and preexisting CVD [14, 15]. But in these international studies, the general population is often used as the reference, which may alter complication rates due to rising diabetes prevalence while simultaneous decline in CVD risk [14]. While most studies on temporal trends in diabetes‐related complications originate from industrialized countries [18, 19], no nationwide study has been conducted in Germany. This highlights the need for detailed analyses of trends in the incidence of AMI, CHD, TIA, and stroke among individuals with incident T2D. Germany's large representative whole population‐linked Disease Analyzer dataset provides an opportunity to address this question.

Our study focuses exclusively on German patients with new‐onset T2D, enabling a precise assessment of temporal changes in CVD incidence and the potential impact of advancements in diabetes care. By excluding nondiabetic populations, we minimize confounding factors, such as shifts in general lifestyle behaviors, ensuring that observed trends are directly attributable to diabetes‐specific interventions [18].

Taken together, the objective of this study is threefold: (i) to assess the 5‐year cumulative incidence and time to onset of MI, CHD, IS, and TIA in a nationwide cohort of patients with new‐onset T2DM from 2001 to 2018 in Germany; (ii) identify temporal trends in the cumulative incidence of these outcomes between 2001–2006 and 2013–2018; and (iii) evaluate key demographic and clinical factors associated with CVD. This research will assist in informing clinical practice and public health strategies for improving cardiovascular outcomes in new‐onset T2D.

## 2 Methods

### 2.1 Database

This retrospective cohort study utilized data from the Disease Analyzer database (IQVIA), a resource that has been extensively used in prior research on diabetes mellitus [20, 21]. The database contains anonymized medical data, including diagnoses and prescriptions, collected from office‐based general practices through their computer systems [22]. It encompasses data from approximately 3000 office‐based general practices across Germany, with the number of participating practices increasing over time. The Disease Analyzer database employs a sampling method based on statistics from the German Medical Association to design its panel according to physician specialization, federal state, community size, and physician age. Previous studies have validated the representativeness of this database for general and specialized practices in Germany [22].

### 2.2 Study Population

**FIGURE 1:**
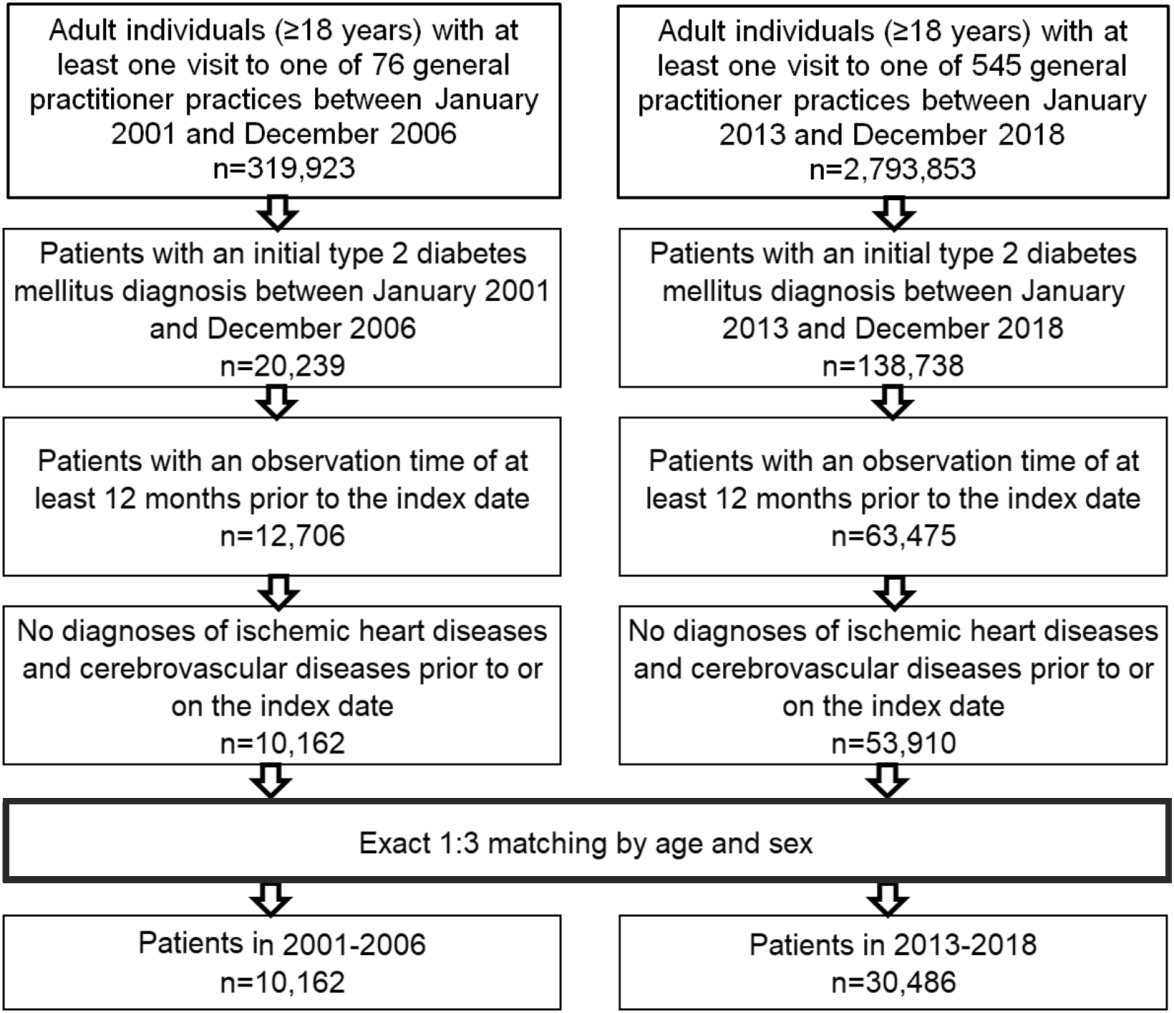
Selection of study participants for the time period 2001–2006 and 2013–2018.

The study included patients aged ≥ 18 years with an initial diagnosis of T2D documented in general practices in Germany during two time periods: January 2001 to December 2006 and January 2013 to December 2018 (index date; see Figure 1). To be included, patients were required to have an observation period of at least 12 months prior to the index date. Patients with prior or concurrent diagnoses of ischemic heart disease (ICD‐10: I20‐I25) or cerebrovascular diseases (ICD‐10: G45, I60‐I69) were excluded. To ensure comparability, patients diagnosed with T2D between 2001 and 2006 were matched 1:3 to patients diagnosed between 2013 and 2018, based on age and sex, using the same inclusion criteria (Figure 1).

### 2.3 Study Outcomes

The outcomes of the study were the initial diagnoses of MI (ICD‐10: I21), CHD (ICD‐10: I25), TIA (ICD‐10: G45), and IS (ICD‐10: I63, I64) within up to 5 years following the first diagnosis of T2D. These outcomes were analyzed as a function of the study period (2013–2018 vs. 2001–2006). Hemorrhagic stroke was infrequently documented and therefore excluded from separate analysis.

### 2.4 Statistical Analyses

Descriptive statistics were presented as means with standard deviations (SDs), medians with interquartile ranges (IQR), or counts with percentages, as appropriate. The 5‐year cumulative incidence of MI, CHD, TIA, and IS during the periods January 2001–December 2006 and January 2013–December 2018 was estimated using Kaplan–Meier survival curves. Patients were followed for up to 5 years from their index date, with data availability extending to December 2023. Multivariable Cox regression models were employed to assess the relationship between the study period and the outcomes. The models were adjusted for chronic comorbidities documented within 12 months prior to the index date, includingobesity (ICD‐10: E66), dyslipidemia (ICD‐10: E78), hypertension (ICD‐10: I10), peripheral arterial disease (PAD; ICD‐10: I70.2, I73.9), and chronic obstructive pulmonary disease (COPD; ICD‐10: J44). COPD was included as a proxy for smoking status due to its strong association with smoking. The regression analyses were stratified by four age groups and conducted separately for male and female individuals. Results from the Cox regression models are presented as hazard ratios (HRs) with 95% confidence intervals (CIs). A *p*‐value of < 0.001 was considered statistically significant due to multiple comparisons to account for the potential inflation of Type I error [23, 24]. Analyses were conducted using SAS version 9.4 (SAS Institute, Cary, USA).

## 3 Results

### 3.1 Baseline Characteristics of the Study Cohorts With New‐Onset T2D Between 2001–2006 and 2013–2018

After 1:3 matching for age and sex, a total of 10 162 patients with a first time T2D diagnosis in 2001–2006 and 30 486 patients with a first time T2D diagnosis in 2013–2018 were included in this study (Figure 1). The demographic characteristics of the study cohorts are displayed in Table 1. The mean age of patients was identical across both cohorts at 62.3 years (SD: 14.4), with no significant differences in the distribution of the five age groups. Approximately 19.7% of patients were aged ≤ 50 years, 21.6% were aged 51–60 years, 29.7% were aged 61–70 years, and 29.0% were aged 70 years or older in both periods. Similarly, the sex distribution was consistent between the cohorts, with 51.9% of patients being women and 48.1% being men. Regarding the comorbidities, there were some notable differences between the two periods. The prevalence of obesity was slightly higher in the 2013–2018 cohort (7.8%) compared to the 2001–2006 cohort (7.0%). Hypertension was also more common in the later cohort, with 43.3% of patients affected compared to 40.4% in the earlier cohort (*p* < 0.001). However, the prevalence of dyslipidemia was similar between the two periods (18.2% in 2001–2006 vs. 17.7% in 2013–2018; *p* = 0.324), as were the rates of PAD (2.1% vs. 2.2%; *p* = 0.828) and COPD (6.2% vs. 6.6%; *p* = 0.144).

**TABLE 1:** Baseline characteristics of new‐onset Type 2 diabetes patients in 2001–2006 and 2013–2018 (after propensity score matching).

Variable	Proportion among patients with T2D in		*p*
	2001–2006	2013–2018	
Participants, *n*	*n* = 10 162	*n* = 30 486	
Women	5273 (51.9)	15 819 (51.9)	1.000
Men	4889 (48.1)	14 667 (48.1)	
Age (mean, SD)			
Total	62.3 [14.4]	62.3 [14.4]	1.000
Age ≤ 50	1999 (19.7)	5997 (19.7)	1.000
Age 51–60	2192 (21.6)	6576 (21.6)	
Age 61–70	3021 (29.7)	9063 (29.7)	
Age 70+	2950 (29.0)	8850 (29.0)	
Comorbidities			
Obesity	710 (7.0)	2.388 (7.8)	0.005
Hypertension	4102 (40.4)	13 187 (43.3)	< 0.001
Dyslipidemia	1846 (18.2)	5406 (17.7)	0.324
PAD	215 (2.1)	656 (2.2)	0.828
COPD	631 (6.2)	2019 (6.6)	0.144

*Note:* Data are presented as *n* (%) unless otherwise indicated.

Abbreviations: COPD, chronic obstructive pulmonary disease; PAD, peripheral arterial disease; SD, standard deviation.

### 3.2 Cardiovascular and Cerebrovascular Complications in New‐Onset T2D Between 2001–2006 and 2013–2018

**FIGURE 2:**
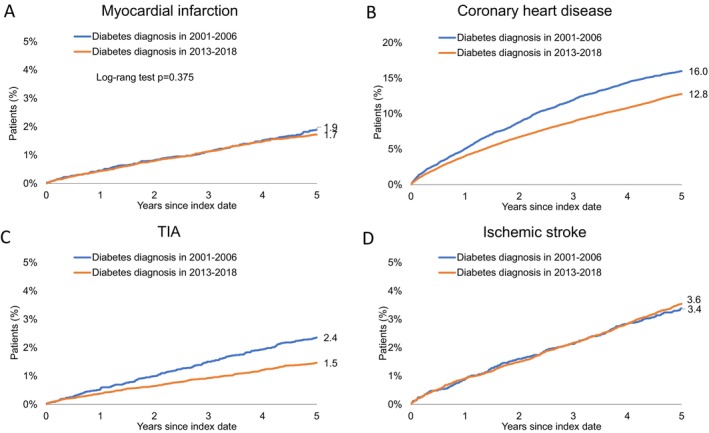
Time trends of the 5‐year cumulative incidence of myocardial infarction (A), coronary heart disease (B), transient ischemic attack (TIA; C) and ischemic stroke (D) in new‐onset Type 2 diabetes patients without prior cardiovascular disease in 2013–2018 versus 2001–2006.

In Figure 2, Kaplan–Meier survival curves for the cumulative incidence of four cardiovascular and cerebrovascular events over the two 5‐year follow‐up periods, 2001–2006 and 2013–2018, for patients diagnosed with new‐onset T2D are shown. The cumulative incidence of MI over 5 years is similar between the two periods, with 1.9% of patients experiencing MI in 2001–2006 compared to 1.7% in 2013–2018 (*p* = 0.375) (Figure 2A). The cumulative incidence of CHD is noticeably lower in the 2013–2018 cohort compared to the 2001–2006 cohort. By 5 years, 16.0% of patients diagnosed in 2001–2006 experienced CHD, compared to 12.8% in the 2013–2018 group (*p* < 0.001) (Figure 2B). The cumulative incidence of TIA is significantly reduced in the 2013–2018 cohort compared to 2001–2006. Over 5 years, 2.4% of patients diagnosed with T2D in 2001–2006 experienced TIA, whereas only 1.5% did in the 2013–2018 cohort (*p* < 0.001) (Figure 2C). The cumulative incidence of IS remains consistent between the two cohorts, with 3.6% in 2001–2006 and 3.4% in 2013–2018 (*p* = 0.563) (Figure 2D).

### 3.3 Temporal Trends of the Cumulative Incidence of Cardiovascular and Cerebrovascular Outcomes in Patients With New‐Onset Type 2 Diabetes Between 2001–2006 and 2013–2018

Table 2 presents the HRs and CIs for the association between the study period (2013–2018 vs. 2001–2006) and the cumulative incidence of cardiovascular and cerebrovascular outcomes in patients with newly diagnosed T2D, adjusted for comorbidities including obesity, hypertension, dyslipidemia, PAD, and COPD.

**TABLE 2:** Temporal changes of myocardial infarction, coronary heart disease, transient ischemic attack, and ischemic stroke incidence cross age and sex subgroups in new‐onset Type 2 diabetes patients (2013–2018 vs. 2001–2006).

Outcome and patient subgroup	HR (95% CI)^a^	*p*
Myocardial infarction		
Total	0.82 (0.68–0.99)	0.045
Age ≤ 50 years	0.85 (0.48–1.53)	0.590
Age 51–60 years	1.10 (0.72–1.68)	0.670
Age 61–70 years	0.65 (0.47–0.97)	0.013
Age 70+ years	0.85 (0.62–1.16)	0.308
Women	0.79 (0.58–1.07)	0.129
Men	0.85 (0.67–1.08)	0.178
Coronary hearth disease		
Total	0.68 (0.63–0.73)	< 0.001
Age ≤ 50 years	0.72 (0.56–0.92)	0.008
Age 51–60 years	0.71 (0.61–0.84)	< 0.001
Age 61–70 years	0.68 (0.60–0.77)	< 0.001
Age 70+ years	0.64 (0.57–0.72)	< 0.001
Women	0.57 (0.51–0.63)	< 0.001
Men	0.79 (0.71–0.87)	< 0.001
Transient ischemic attack		
Total	0.63 (0.52–0.76)	< 0.001
Age ≤ 50 years	1.46 (0.60–3.55)	0.403
Age 51–60 years	0.70 (0.43–1.16)	0.167
Age 61–70 years	0.58 (0.41–0.82)	0.002
Age 70+ years	0.57 (0.43–0.75)	< 0.001
Women	0.58 (0.45–0.75)	< 0.001
Men	0.69 (0.52–0.92)	0.013
Ischemic stroke		
Total	0.97 (0.85–1.12)	0.722
Age ≤ 50 years	1.33 (0.70–2.51)	0.379
Age 51–60 years	1.51 (1.00–2.28)	0.049
Age 61–70 years	0.99 (0.76–1.29)	0.950
Age 70+ years	0.82 (0.67–0.99)	0.044
Women	0.94 (0.77–1.15)	0.551
Men	1.01 (0.82–1.23)	0.949

^a^ Adjusted for obesity, hypertension, dyslipidemia, peripheral arterial disease (PAD) and chronic obstructive pulmonary disease (COPD).

For MI, the overall HR in the 2013–2018 cohort compared to the 2001–2006 cohort was 0.82 (95% CI: 0.68–0.99; *p* = 0.045), indicating a trend toward a lower cumulative incidence. A notable trend of reduction was also observed in the 61–70‐year age group (HR: 0.65; 95% CI: 0.47–0.97; *p* = 0.013), but no significant differences were found in any age group or by sex.

In contrast, coronary heart disease showed a significant overall reduction in cumulative incidence, with an HR of 0.68 (95% CI: 0.63–0.73; *p* < 0.001), consistent across all age groups. The largest reduction was observed in patients aged 70+ years (HR: 0.64; 95% CI: 0.57–0.72; *p* < 0.001), with a more pronounced reduction in women (HR: 0.57; 95% CI: 0.51–0.63; *p* < 0.001) than in men (HR: 0.79; 95% CI: 0.71–0.87; *p* < 0.001).

The cumulative incidence of TIA was also significantly lower in the 2013–2018 cohort, with an HR of 0.63 (95% CI: 0.52–0.76; *p* < 0.001). A subgroup analysis revealed a significant reduction in patients aged 61–70 years (HR: 0.58; 95% CI: 0.41–0.82; *p* < 0.001) and those aged 70+ years (HR: 0.57; 95% CI: 0.43–0.75; *p* < 0.001), with women showing a stronger reduction (HR: 0.58; 95% CI: 0.45–0.75; *p* < 0.001) than men (HR: 0.69; 95% CI: 0.52–0.92; *p* = 0.013).

For IS, the overall HR in the 2013–2018 cohort compared to the 2001–2006 cohort was 0.97 (95% CI: 0.85–1.12; *p* = 0.722), indicating no significant difference in the cumulative incidence. A trend toward a reduction in the incidence was observed in patients aged 70+ years (HR: 0.82; 95% CI: 0.67–0.99; *p* = 0.044), but this result did not reach statistical significance.

## 4 Discussion

This nationwide German study examining temporal trends in diabetes‐related cardiovascular complications between 2001–2006 and 2013–2018 identified several significant findings. First, the 5‐year cumulative incidence of CHD and TIA declined among individuals with newly diagnosed T2D and no prior CVD. Second, these reductions were more pronounced in older adults and women. Third, the incidence of IS and MI remained stable, likely reflecting the increasing prevalence of prediabetes and diabetes, which may offset improvements in cardiovascular risk management. Hypertension emerged as a key contributor to these trends, underscoring its critical role as a target for preventive interventions.

This study is the first to provide a comprehensive analysis of the temporal development of diabetes‐related cardiovascular complications in Germany. Of note, a direct comparison of incidence rates between studies, even from comparable countries [25, 26, 27, 28], is challenging due to variations in methodology [18], cohort characteristics, and inclusion criteria, such as older patients with more comorbidities and longer disease duration [16, 19], and data sources [29].

Unlike most prior studies, this study focused exclusively on new‐onset T2D (duration < 12 months) without previously known CVD. Nevertheless, the incidence rates reported here are broadly consistent with recent studies from England [8], Denmark [30] and other countries [25, 28, 31, 32], which have shown a continuous decline in cardiovascular complications in individuals with long‐standing T2D (> 5 years) in comparison to the general population.

Consistent with this, we demonstrate a declining 5‐year cumulative incidence rate of CHD and TIA in individuals with new‐onset T2D without prior CVD in Germany over a 17‐year period. This decline is particularly noteworthy given the clinical importance of TIA as a precursor to cerebral infarction [33] and vascular mortality [34]. Diabetes is known to increase the risk of TIA by 50% [35] and increase the likelihood of recurrent TIA and subsequent IS, highlighting its importance in cerebrovascular risk stratification. In addition, this study presents the first analysis of temporal trends in TIA incidence in T2D populations in Germany and worldwide.

In contrast, the 5‐year cumulative incidence of MI in individuals with new‐onset T2D and no previous CVD remained stable, diverging from the majority of prior literature [8, 14], although some exceptions exist [36]. A parallel observation was made in the incidence of first‐time IS, which remained stable at 3.4% in 2001–2006 and 3.6% in 2013–2018 among individuals with new‐onset T2D, as seen before [26].

The observed reduction in CHD and TIA suggests advancements in diabetes care, including earlier diagnosis, improved risk factor control, and lifestyle interventions. However, the stable rates of MI and IS may indicate that significant vascular damage occurs long before diabetes onset [37]. Prediabetes is increasingly recognized as a state of heightened cardiovascular risk rather than merely a transition phase toward diabetes. Previous research has demonstrated that individuals with prediabetes already exhibit atherogenic lipid patterns, endothelial dysfunction, and early markers of vascular damage [38]. Notably, even individuals with high‐normal glucose tolerance during an oral glucose tolerance test have been shown to exhibit a cardiometabolic risk profile similar to those with impaired glucose tolerance [38]. Given that over one‐third of adults have often undiagnosed prediabetes, this prolonged exposure to dysglycemia and insulin resistance could contribute to the pathogenesis of MI and IS risk [39]. Thus, the stable MI and IS incidence observed in this study may be a consequence of cardiovascular risk accumulating before T2D is diagnosed. While advances in diabetes management over the last two decades, such as earlier diagnosis, improved blood pressure and lipid control, and widespread statin use [11, 12, 26, 40], have contributed to a decline in CHD and TIA, these same interventions may not be sufficient to offset the vascular damage that accumulates over years of prediabetes. This may explain why CHD rates declined, but MI incidence remained largely unchanged. Unlike CHD, which includes chronic ischemic conditions, MI is often triggered by acute plaque rupture, potentially reflecting long‐standing atherosclerotic burden that developed before diabetes onset. Similarly, TIA declined while IS remained stable, which may be attributed to improved early detection and management of transient cerebrovascular events but persistent arterial damage caused by long‐term dysglycemia. In line, a recent population‐based study from Denmark highlighted the persistently elevated 10‐year CVD risks in newly diagnosed T2D patients, particularly among younger individuals below 49 years, compared to the general population [41]. Much of this elevated risk may stem from prediabetic factors, as T2D patients experience twice as many CVD events as non‐diabetics, starting as early as three decades before diagnosis and highlighting that CVD and thus myocardial infarction is a simultaneous pathogenesis to development of T2D [27].

Furthermore, it is imperative to consider the following: the majority of preceding population‐based studies have evaluated incidence rates in relation to the general population as a whole, rather than exclusively within the context of diabetes populations. This is exemplified by analyses from Sweden, Scotland, the United States, and South Korea [19, 28, 32, 42, 43]. The reported decline in cardiovascular complications among individuals with long‐standing T2D (duration > 5 years) has been paralleled by improvements in glycemic control, cardiovascular risk factors, and reduced prevalence of hypertension, dyslipidemia, and smoking, especially in the general population [16, 44, 45, 46]. The interpretation of trends in diabetes complications is influenced by the choice of denominator population. Comparisons to the general population may obscure trends within the diabetes population, as rising diabetes prevalence and increasing CVD burden in the general population can distort the observed trajectories of diabetes complications [14, 19]. A Swedish cohort study demonstrated stable rates of acute MI and IS from 2003 to 2008 despite a growing diabetes population, suggesting that the increasing prevalence of T2D may offset expected declines in cardiovascular event rates [43]. Comparisons between studies are further complicated by differences in cardiovascular risk profiles between newly diagnosed and chronic T2D populations, as well as evolving patterns in disease presentations, which were beyond the scope of this study [18]. Thus, the decline in CHD and TIA likely reflects improvements in early diagnosis, risk factor control, and preventive interventions, whereas the stable incidence of MI and IS suggests that significant vascular damage accumulates during the prediabetic phase, limiting the impact of later interventions.

Another important finding of this study is the critical influence of age and sex on the burden of diabetes‐related complications, with evidence of shifting dynamics over time. Between 2001–2006 and 2013–2018, significant reductions in CHD incidence were observed starting from age 50 and in TIA incidence from age 70, with more pronounced improvements among women. Notably, age‐period‐cohort effects were more evident in younger individuals, aligning with patterns increasingly documented in the diabetes‐related complication literature [25, 29, 47] and extending to mortality trends [16]. While these observations might suggest disparities in diabetes care by age, they do not fully explain the temporal changes. A more plausible explanation could be the greater impact of recent socioeconomic conditions on younger individuals [47], although this phenomenon remains poorly understood. The absence of improvement may be attributable to the rising prevalence of hypertension as a diabetes‐related risk factor in younger populations [48]. Another factor influencing these trends may be the increased survival of individuals with diabetes, evident in the rising median age and longer duration of diabetes, which provides more time for complications to manifest [25]. Our findings show rising event counts over time, reflecting the increasing diabetes prevalence and underscoring the urgent need for effective prevention and management strategies.

This study has several strengths, including the use of a nationwide, population‐based dataset from a tax‐supported healthcare system, minimizing selection bias and ensuring near‐complete follow‐up. Validation studies confirm the high positive predictive value of ICD‐10 codes for identifying the diabetes population and study outcomes [25, 49]. The dataset's size and representativeness allowed for robust estimates of trends in hospitalizations for major diabetes‐related complications across all age groups with incident T2D [22]. However, limitations must be acknowledged. The retrospective cohort design relies on preexisting data, introducing potential selection bias. The dataset may not fully capture all patient populations or uniformly represent variables across practices. While data from general practitioners excludes patients exclusively managed in hospitals, severe complications are generally reported back to general practitioners for follow‐up care. The Disease Analyzer Database lacks laboratory data, including HbA1c, and critical clinical variables such as estimated glomerular filtration rates and lifestyle factors (e.g., smoking, diet, physical activity), which are key contributors to ASCVD risk. COPD was used as a proxy for smoking but does not account for temporal changes in smoking prevalence, such as its decline in Germany. The reliance on ICD‐10 codes, though highly accurate for identifying acute events [32, 49], may introduce inconsistencies due to variations in provider documentation practices. Newly included practices in later cohorts might document conditions differently, potentially affecting trend interpretations. Despite these limitations, the use of standardized ICD‐10 codes enhances the generalizability of findings across healthcare systems, while the longitudinal design enables insights into trends over time, contributing to a deeper understanding of improvements in diabetes care.

This study provides the first comprehensive analysis of temporal trends in diabetes‐related complications in Germany, examining new‐onset T2D without prior CVD from 2001–2006 and 2013–2018. While CHD and TIA incidence declined—particularly, among older adults and women—MI and IS rates remained stable, underscoring persistent challenges in CVD prevention and the long‐term vascular impact of prediabetes. These findings highlight the need for early, targeted diagnostics and interventions tailored to age‐ and sex‐specific subgroups, as well as improved prediabetes management to reduce the growing burden of diabetes complications on individuals and healthcare systems.

## Ethics statement

Ethical review and approval were waived for this study as strictly registry‐based studies do not require ethical approval or informed consent from participants. This was approved by the ethics committee of Heinrich‐Heine‐University. Patient data were analyzed in aggregated form without individual data being available.

## Consent

Individual consent forms were not required or obtained, in accordance with national and European legislation.

## Conflicts of Interest

The authors declare no conflicts of interest.

## Data Availability

The datasets used and analyzed during the current study are available from the corresponding author on reasonable request.
